# Molecular Diagnosis of Thyroid Nodules Using Next-Generation Sequencing in the Chinese Population

**DOI:** 10.1155/ije/7728360

**Published:** 2025-06-20

**Authors:** Hui Chen, Wei Liu, Yan Chen, Zhengzeng Jiang, Yuanyuan Ren, Jiajia Wu, Rui Liu, Min Zhu, Hongfeng Zhang, Yuan Ji

**Affiliations:** ^1^Department of Ultrasonic Diagnosis, The First Hospital of Putian City, Putian 351100, China; ^2^Xiangya Medical Laboratory, Central South University, Changsha 410013, China; ^3^Department of Pathology, Zhongshan Hospital, Fudan University, Shanghai 200032, China; ^4^Singlera Genomics (Shanghai) Ltd., Shanghai 201315, China; ^5^Department of Pathology, The Central Hospital of Wuhan, Wuhan 430014, China

**Keywords:** diagnostic performance, DNA–RNA test, mutation profiling, next-generation sequencing, thyroid nodules

## Abstract

**Background:** Fine-needle aspiration (FNA) cytology remains a challenge in the diagnosis of indeterminate thyroid nodules. Molecular testing can bridge the gap left by FNA cytology and improve the diagnostic accuracy of FNA.

**Methods:** 786 FNA samples and 40 formalin-fixed paraffin-embedded (FFPE) specimens from thyroid nodules were enrolled in next-generation sequencing (NGS) molecular testing, which included gene mutation and gene fusion analysis. The molecular diagnostic performance was assessed by analyzing sensitivity, specificity, accuracy, negative predictive value (NPV), and positive predictive value (PPV).

**Results:** Among 826 thyroid nodules, 409 were NGS-positive (49.52%), with a high prevalence of *BRAF* V600E (36.32%, 300/826) and *RAS* (9.32%, 77/826) mutations, a low prevalence of *TERT* promoter mutations (1.69%, 14/826), and gene fusions involving *RET* (1.82%, 15/826), *NTRK3* (0.73%, 6/826), *ALK* (0.24%, 2/826), and *PAX8-PPARG* (0.12%, 1/826). With the analysis of genetic profiles in thyroid nodules, *BRAF* V600E, *TERT* mutations, and gene fusions were included in the 6-gene test panel. The overall diagnostic performance of the 6-gene test panel, including sensitivity, specificity, accuracy, NPV, and PPV, was 84.87%, 89.61%, 86.26%, 71.13%, and 95.15%, respectively. For thyroid nodules in Bethesda III, IV, and V, the diagnostic sensitivity, specificity, accuracy, NPV, and PPV of the panel were 85.71%, 88.89%, 86.36%, 61.54%, and 96.77%, respectively.

**Conclusion:** The results reveal that the 6-gene test panel as a “rule in” test in a clinical setting improves the accuracy of FNA cytology, potentially assisting in the diagnosis of the thyroid nodules with indeterminate FNA cytology.

## 1. Introduction

Thyroid cancer is the most common endocrine malignant tumor worldwide and is rapidly increasing with an estimated occurrence of 220,000 cases in China by 2020 [1]. The most recent global cancer data indicate a significantly higher incidence of thyroid cancer in women compared to men, with an average annual increase of approximately 20%. Thyroid cancer typically manifests in patients with thyroid nodules. High-quality thyroid ultrasonography (US) and fine-needle aspiration (FNA) biopsy are widely utilized as the primary and dependable diagnostic modalities for distinguishing benign nodules from tumors. However, approximately 25% of nodules cannot be definitively excluded as cancer by FNA, posing a potential dilemma for both patients and clinicians [2]. Similar to other malignancies, the emergence and advancement of thyroid cancer are accompanied by genetic mutations, such as *RAS*, *BRAF*, or *TERT* mutations, as well as *RET* and *NTRK* rearrangements [3, 4]. Consequently, the incorporation of molecular testing serves as an additional method to complement the limitations of FNA cytology and assists in the identification of malignant thyroid nodules. It is recommended by the guidelines of the Chinese Society of Clinical Oncology and the American Thyroid Association [2].

Multiple genetic mutations identified in thyroid cancer are receiving increasing attention, including *BRAF*, *RAS*, *TERT*, *PIK3CA*, *AKT*, *PTEN*, *RET*, *TP53*, and gene fusions of *RET* and *NTRK* [5, 6]. Notably, *BRAF* V600E mutations are the most prevalent and significant, accounting for 98∼99% of all the *BRAF* mutations, which occur in approximately 40∼70% of papillary thyroid cancer (PTC) and 30∼40% of anaplastic thyroid cancer, especially in Chinese patients [3, 7, 8]. A study conducted on a cohort of the Chinese population revealed that the frequency of the *BRAF* V600E mutation in PTC exceeds 75%, which is higher than the rates observed in Indonesian and French populations [9]. The prevalence of *RAS* mutations is second only to *BRAF* mutations in differentiated thyroid cancer. *RAS* mutations are found not only in malignant tumors but also in benign thyroid nodules [10, 11]. The *TERT* gene encoding the catalytic subunit of telomerase is closely associated with tumorigenesis and development, as its activation plays a significant role in these processes [12, 13]. *RET* fusion and *NTRK* fusion are the most common rearrangements observed in thyroid cancer [14, 15]. In cases of thyroid cancer, the predominant fusion events involve *RET/PTC1* and *RET/PTC3*, which entail the fusion of the *RET* gene with the *CCDC6* gene and the *NCOA4* gene, respectively [16]. The presence of *ETV6-NTRK3* is linked to the tumor size, metastasis, and patient prognosis [17, 18].

Molecular alterations in thyroid nodules, as described above, have been identified as useful diagnostic and prognostic markers for thyroid cancer. Large-scale studies have fully elucidated the genomic and pathological changes in thyroid cancer, particularly thyroid follicular carcinoma, in Western populations [19]. Multiple gene panels for thyroid FNA samples are available in the United States, including ThyroSeq V3, Afirma GSC, and ThyraMIR [20]. The reported data indicate differences in genetic mutations associated with thyroid cancer between Chinese and Western patients. For instance, the *BRAF* V600E mutation exhibits higher frequencies in the Chinese population [8, 21]. However, the prevalence of *RAS* mutations in PTC is lower in the Chinese population compared to the data from the TGCA study (1% vs. 13%) [22, 23]. There are limited studies on the mutation profiles in thyroid nodules specific to the Chinese population.

In the present study, the 22-gene mutation assay (*AKT1*, *APC*, *ATM*, *BRAF*, *CTNNB1*, *EIF1AX*, *GNAS*, *HRAS*, *KRAS*, *NRAS*, *PIK3CA*, *PTEN*, *RET*, *TG*, *TP53*, *TSHR*, *TERT*, *TTN,* as well as gene fusions involving *NTRK1*, *NTRK3*, *ALK*, *PPARG,* and *RET*) was utilized to detect gene mutations and fusions associated with thyroid cancer in a substantial cohort of 826 samples obtained from FNA samples and surgical formalin-fixed paraffin-embedded (FFPE) specimens. This study aimed to delineate the genetic landscape of thyroid nodules in China, providing deep insights into disease formation and helping to stratify patients according to their risk level for malignancy. Furthermore, it was intended to evaluate the diagnostic and screening performance of NGS assays for thyroid cancer in FNA samples, particularly in the case of indeterminate nodules and suspicious malignancy (SUSP) nodules.

## 2. Materials and Methods

### 2.1. Patients and Clinical Specimens

A total of 826 patients with thyroid nodules were enrolled in the study conducted from May 2022 to July 2023. Of these, 736 samples were collected from The First Hospital of Putian City, and another 90 samples were from Zhongshan Hospital. All participants in the study provided informed consent, and the research protocol was approved by the Institutional Ethics Committee of The First Hospital of Putian City (No 2022-032) and the Institutional Ethics Committee of Zhongshan Hospital (No B2022-609R). Written informed consent was obtained from each individual who participated in the study. The sample types used for molecular testing included 786 FNA samples and 40 FFPE specimens. The FNA procedure was conducted under ultrasound guidance by an experienced thyroid surgeon or endocrinologist/radiologist, following a standardized protocol of each institution.

### 2.2. Nucleic Acid Isolation

Genomic DNA/RNA of FNA samples was extracted using the nucleic acid extraction kit (Shanghai Singlera, China), and FFPE samples were processed using the FFPE extraction kit (CoWin Biotech, China). The elution volumes for DNA/RNA were 30 μL. The purity of DNA/RNA was assessed using a UV spectrophotometer (NanoDrop Technologies, Thermo Fisher Scientific Inc, USA), and quantification was conducted with Qubit 3.0 (Thermo Fisher Scientific Inc, USA).

### 2.3. Library Preparation

The sequencing libraries were prepared using the OncoAim thyroid cancer multigene assay kit (Singlera Genomics, Inc., Shanghai, China). RNA fusion was detected based on cDNA, and SNVs/indels were detected based on DNA. After PCR and purification, the barcodes and adapters specific to GENETRON S5 based on semiconductor sequencing technology (Genetron, China) were incorporated, and the quality of the prepared libraries was assessed using the LabChip GX Touch24 (PerkinElmer, USA). The sequencing depth is above 1000X. The sequencing procedure was conducted in accordance with the reagent protocols using the GENETRON S5.

### 2.4. Sequencing Data Analysis

Raw reads were filtered and mapped to the reference human genome (UCSC, hg19) using the GENETRON S5 platform. Subsequently, the sequencing quality was assessed. The sequencing data quality control was considered acceptable if the average coverage was ≥ 1000, the uniformity was ≥ 70%, and the coverage of the target regions was ≥ 95%. For DNA analysis, the Freebayes (v1.0.2) was used for SNVs/indels calling. The identified variants were annotated using the variant effect predictor (VEP, v90.9) in conjunction with the dbSNP (v152) and COSMIC (v91) databases. For RNA analysis, blast (v2.2.20) was used for gene fusion calling and analysis. This 22-gene mutation assay has the capability to detect single-nucleotide variants (SNVs) and insertions/deletions (Indels) in the *AKT1*, *APC*, *ATM*, *BRAF*, *CTNNB1*, *EIF1AX*, *GNAS*, *HRAS*, *KRAS*, *NRAS*, *PIK3CA*, *PTEN*, *RET*, *TG*, *TP53*, *TSHR*, and *TTN*, as well as variants in the *TERT* promoter region (C228T, chr5:1295228C>T, and C250T, chr5:1295250C>T), the *NTRK1*, *NTRK3*, *ALK*, *PPARG,* and *RET* fusion variants simultaneously.

### 2.5. Interpretation of Molecular Testing Results

The molecular testing results were categorized into two components: gene mutation and gene fusion. Positive identification was made for the *BRAF* V600E gene mutation with a frequency of ≥ 0.2%, and mutation-positive status was determined for other genes (*AKT1*, *APC*, *ATM*, *BRAF*, *CTNNB1*, *EIF1AX*, *GNAS*, *HRAS*, *KRAS*, *NRAS*, *PIK3CA*, *PTEN*, *RET*, *TG*, *TP53*, *TSHR*, *TERT*, and *TTN*) with a mutation frequency of ≥ 2%. The presence of *NTRK1*, *NTRK3*, *ALK*, *PPARG*, or *RET* gene fusion mutations with a frequency of CP100K ≥ 500 was determined to be positive (CP100K: average number of reads of fusion gene sequences per 100 K total number of reads).

### 2.6. Statistics

Patient age was compared using a *t*-test. Patient gender and nodule size were analyzed using chi-square test or Fisher's exact test. *p* < 0.05 was considered statistically significant. Sensitivity, specificity, positive predictive value (PPV), and negative predictive value (NPV) were calculated with 95% Wilson confidence intervals. The area under the curve (AUC) was computed by MEDCALC v18.2.1 (MedCalc Software, Ostend, Belgium).

## 3. Results

### 3.1. Basic Information on Samples

This study involved the analysis of 786 FNA samples and 40 FFPE specimens through gene sequencing, comprising 684 samples obtained from female patients and 142 samples from male patients (Table 1). In this cohort, the ratio of females to males was 4.82, and the average age at diagnosis was 48.98 years (with a range of 15–82 years). The overall incidence is higher in women than in men, consistent with previous reports [24]. The patients' ages were categorized into youth (14–47 years), middle-aged (48–63 years), and old (> 64 years). Cytologically, 198 (23.97%) patients were not obtained for FNA results, categorized as no cytology. 45 samples were confirmed as Bethesda I, primarily due to insufficient or inadequate tissue (Table 2). Additionally, 250 (30.27%) samples were diagnosed as benign, 187 (22.64%) were classified as indeterminate, 129 (15.62%) were deemed suspicious for malignancy, and 17 (2.06%) were identified as malignant. A total of 524 surgical histopathological specimens were not collected as a result of a decision without surgery, or surgical scheduling. 77 specimens were surgically determined to be benign, while 225 specimens were confirmed to be malignant tumors.

### 3.2. The Landscape of Molecular Alterations in NGS Results

**Figure 1:**
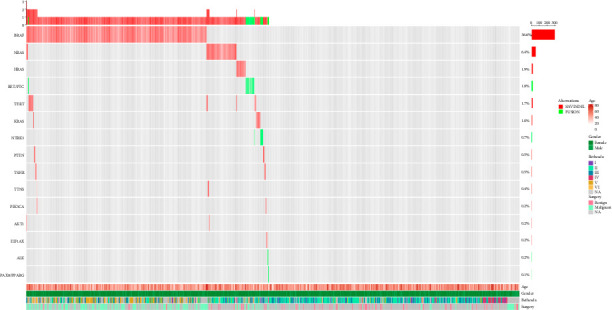
Mutation profiling of all samples by NGS testing. Clinicopathological features included age, gender, cytology, and surgical pathology. Mutated genes included *BRAF*, *RET*, *TERT* promoter, *NRAS*, *KRAS*, *HRAS*, *NTRK3*, *PTEN*, *TSHR*, *TTNS*, *PIK3CA*, *AKT1*, *EIF1AX*, *ALK*, and *PAX8/PPARG*.

The 22-gene mutation assay was performed to investigate the genetic alterations in the thyroid nodules. Molecular testing for RNA and DNA was conducted simultaneously on all 826 samples that contained a sufficient amount of both DNA and RNA. The results of the testing indicated that 417 samples were categorized as negative, while 409 samples exhibited genetic variants within the detectable range, resulting in a positive rate of 49.52% (Figure 1 and Tables 3 and 4). The NGS-positive patients were significantly younger than the NGS-negative patients (47.56 vs. 50.37 years, *p* < 0.01). No significant relationship was observed with respect to gender (*p*=0.770) (Table 3). The most prevalent mutation observed was *BRAF* V600E, with 300 (73.35%) samples classified as positive, and two samples carried the rare *BRAF* K601E mutation. The *BRAF* V600E-positive patients were also significantly younger than the *BRAF* V600E-negative patients (46.95 vs. 50.13 years, *p* < 0.001). Similarly, no significant relationship was observed regarding gender (*p*=0.785) (Table 3). *RAS* mutations were the second most common mutations, with a prevalence of 18.83% (77/409). *NRAS* mutations were detected in 53 cases, occupying the most prevalence of *RAS* mutations, followed by *HRAS* mutations (*n* = 16), and the least by *KRAS* mutations (*n* = 8) (Table 4). Moreover, *RAS* mutations were found in patients with malignant tumors (*n* = 15) and benign tumors (*n* = 9). 14 samples exhibited *TERT* mutations (1295228C>T, 1295250C>T), with a prevalence of 3.42%. The *TERT* promoter mutation-positive patients tended to be older than the mutation-negative patients (62.07 vs. 48.75 years, *p* < 0.01), with no significant relationship observed concerning gender (*p*=0.075) (Table 3). Gene fusions emerged as another common type of mutation, with 15 (3.67%) samples confirmed to exhibit *RET/PTC* fusion (*CCDC6-RET* and *NCOA4-RET*), followed by *NTRK3* fusion (*ETV6-NTRK3* and *EML4-NTRK3*) with 6 (1.47%) samples. Some rare gene fusions were found in this cohort, including *STRN-ALK* (*n* = 2) and *PAX8-PPARG* (*n* = 1). Additionally, the gene fusion-positive patients tended to be younger than the gene fusion-negative patients (43.79 vs. 49.13 years, *p* < 0.05), with no significant relationship noted with respect to gender (*p*=0.784) (Table 3). No statistically significant differences were observed in mean nodule size between the nodules with genetic alterations and those without, including *BRAF* V600E mutations, *TERT* promoter mutations, and gene fusions (Table S1). Four samples carried *PTEN* mutations, with two having *PTEN* mutations only and two having *BRAF* V600E and *PTEN* mutations both. Four samples with *TSHR* mutations (two with *TSHR* only and two with *BRAF* V600E/*TSHR*) were confirmed to be one malignant tumor, one benign tumor, and two lack of surgical pathology. *TTN* S3536N mutations were detected in three patients, but no surgical pathology was performed. Two samples with *PIK3CA* mutations (one with *PIK3CA* only and one with *BRAF* V600E/*PIK3CA*) were confirmed to be one malignant tumor and one benign tumor. Two samples carried only *EIF1AX* mutations.

### 3.3. Genetic Features in Different Subtypes of Thyroid Carcinomas

**Figure 2:**
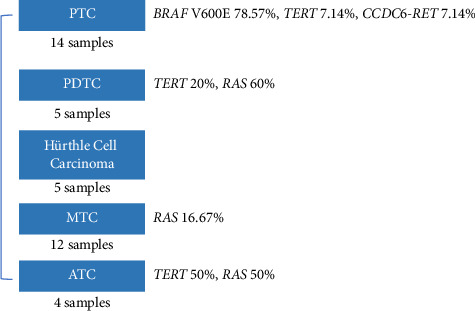
Genetic alterations of different thyroid carcinomas in FFPE samples. PTC, papillary thyroid carcinoma; PDTC, poorly differentiated thyroid carcinoma; MTC, medullary thyroid carcinoma; ATC, anaplastic thyroid carcinoma.

40 FFPE samples included 14 identified as papillary thyroid carcinomas (PTCs), 5 poorly differentiated thyroid carcinomas (PDTCs), 5 Hurthle cell tumors, 4 anaplastic thyroid carcinomas (ATCs), and 12 medullary thyroid carcinomas (MTCs). The results of the NGS testing indicated that 20 samples were categorized as positive. The detailed sequencing results of the whole cohort are described in supporting information (Table S2). Different gene mutations were observed in different subtypes of thyroid carcinomas (Figure 2). PTC is the most common subtype, accounting for approximately 35% of the FFPE cases. 11 samples exhibited *BRAF* V600E, with a prevalence of 78.57% in PTC. Mutations were not detected in 2 PTC samples. The most prevalent mutation observed in PDTC was RAS, with a prevalence of 60%. Moreover, one carried *TERT* C228T, and two PDTCs were categorized as negative. However, genetic alterations were not detected in 5 Hurthle cell tumors. Another subtype at a low mutation frequency was MTC, with only 2 samples carrying *RAS* mutations. In 4 ATCs, the prevalence of TERT mutation and RAS mutation was 50%.

### 3.4. Samples With Two or More Mutations

The NGS profiles of thyroid nodules indicated that the majority of NGS-positive cases exhibited single-gene mutations (*n* = 385), whereas a few cases had multiple genetic alterations (*n* = 24). 24 samples exhibited the presence of two or three mutations. Eight samples were found to carry the *BRAF* V600E and *TERT* co-mutations. 6 cases exhibited the *BRAF* V600E and *TERT* C228T co-mutations, while two cases showed the *BRAF* V600E and *TERT* C250T co-mutations. Six of the samples were confirmed to be malignant tumors through surgical pathology and were specifically identified as PTC (Table 5). The other two cases were categorized by FNA cytology as Bethesda II and V, respectively. However, they lack the pathological results and follow-up. Patient FNA-0002 exhibited the *BRAF* V600E mutation combined with *CCDC6-RET* fusion and was categorized as Bethesda V based on FNA cytology. She also lacked the results of surgical pathology. Patient FNA-0480 also presented with the *TERT* C228T mutation and *ETV6-NTRK3* fusion, as confirmed by surgical pathology indicating benign conditions. Three samples were identified as carrying *BRAF* V600E and *RAS* mutations (two with *BRAF* V600E/*NRAS* and one with *BRAF* V600E/*KRAS*), and of them, patient FNA-0692, with *BRAF* V600E and *NRAS* Q61R, was confirmed as PTC by surgical pathology. In addition to the *BRAF* V600E mutation, *TERT* mutations were the second most common among multiple mutations. Two samples carried the *TERT* C228T and *RAS* mutations, and one (FFPE-026) was confirmed as PDTC with the invasion of nerve bundles by histopathology, and the other was categorized as Bethesda IV by cytology. According to sequencing analysis, *AKT1* mutations were significantly associated with the presence of *NRAS* mutations (one with *BRAF* V600E/*NRAS* Q61R/*AKT1* and one with *NRAS* Q61R/*AKT1*). Patient FNA-0330, who has three mutations (*BRAF* V600E/*NRAS* Q61R/*AKT1*), was confirmed as follicular thyroid carcinoma (FTC) with lymph node metastasis. Patient FFPE-035 with three mutations (*TERT* C228T/*TERT* C250T/*NRAS* Q61R) was confirmed as FTC.

In addition to the samples with a lack of surgical pathology, 90% of patients with two or three mutations developed malignant thyroid cancer. Hence, an increased number of mutations may indicate a greater probability of malignancy. However, the sample size was insufficient to facilitate a comprehensive analysis and to establish more definitive conclusions.

### 3.5. Diagnostic Performance of the NGS Testing for Thyroid Nodules

The diagnostic performance of the 22-gene mutation assay was calculated. Among the 786 FNA samples, the positive rates of the 22-gene mutation assay were 17.78% (8/45) in nondiagnostic cytology (Bethesda I), 29.20% (73/250) in benign cytology (Bethesda II), 40.11% (75/187) in indeterminate cytology (Bethesda III & IV), 85.27% (110/129) in SUSP cytology (Bethesda V), and 94.12% (16/17) in malignant cytology (Bethesda VI). The positive rate of the 22-gene mutation assay was found to increase in correlation with the risk of cytologic malignancy. Surgical pathology was taken as the gold standard for diagnosing malignancy. In this cohort, 262 FNA samples underwent surgical resections, of which 185 were histologically confirmed as malignant, and 77 samples were identified as benign (Table 6). The diagnostic results of the 22-gene mutation assay revealed a positive rate of 69.85% (183/262) and a negative rate of 30.15% (79/262). The diagnostic performance of 22-gene mutation assay in thyroid nodules revealed 88.11% sensitivity, 74.03% specificity, 72.15% NPV, 89.07% PPV, and an overall accuracy of 83.97% (Table 6). Limitations of several gene mutations for diagnosing thyroid nodules were identified, including the observation of *RAS* mutations in benign thyroid nodules. With the analysis of sequencing results, 147 FNA samples with *BRAF* V600E mutation were confirmed with malignant tumors by surgical pathology and 6 samples were benign. Five FNA samples (83.33%, 5/6) with *TERT* mutations were identified as malignant tumors, while one was benign. 10 FNA samples (83.33%, 10/12) with gene fusions were confirmed with malignant tumors, while two were benign nodules. Therefore, the targets widely recognized and recommended by scholars at home and abroad were selected to compose a 6-gene test panel for thyroid nodule diagnosis, including *BRAF* V600E mutation, *TERT* mutation, and gene fusions (*CCDC6-RET*, *NCOA4-RET*, *ETV6-NTRK3*, *EML4-NTRK3*, *STRN-ALK*, and *PAX8/PPARG*). The diagnostic performance of the 6-gene test panel in detecting thyroid nodules revealed a sensitivity of 84.87%, specificity of 89.61%, NPV of 71.13%, PPV of 95.15%, and an overall accuracy of 86.26% (Table 6).

The major objective of this molecular testing was to distinguish histopathological malignant nodules from cytologically indeterminate nodules, which causes a dilemma in clinical management. In this cohort, the prevalence of indeterminate nodules (Bethesda III, IV) was found to be 23.79% (187/786), of which only 48 samples underwent surgical resections. Upon further analysis of the surgical pathology results in Bethesda V, it was found that only 31.01% (40/129) of patients opted for surgery, with a high malignant detection rate of 100%. Consequently, there was a high demand for molecular testing results for samples categorized as suspicious for malignancy (Bethesda V) and indeterminate nodules, to assist patients in making informed decisions regarding subsequent treatment. When relying on the presence of the 6-gene test panel, 26 negative samples and 62 positive samples were detected (Table 7). The performance of the 6-gene test panel in diagnosing thyroid nodules (Bethesda III, IV, and V) demonstrated a sensitivity of 85.71%, specificity of 88.89%, NPV of 61.54%, PPV of 96.77%, and an accuracy of 86.36%.

## 4. Discussion

In this study, the 22-gene mutation assay demonstrated the ability to detect gene mutations and gene fusions with minimal amounts of DNA and RNA, thereby contributing to the diagnostic process of thyroid cancer. Our results suggested that genetic alterations, including *BRAF* V600E and gene fusions, were associated with younger age, while *TERT* promoter mutations were related to older age. There was no significant association between gender or nodule size and gene mutations. This finding is consistent with the results of Yang et al. regarding the prevalence of *TERT* promoter mutations and their clinicopathological associations in 2092 Korean patients [25]. Chen et al. reported that no significant correlation was observed between average nodule size and *BRAF* V600E mutations [26]. However, some reported literature correlating mutations with gender or age were conflict with our findings. For instance, Sun et al. demonstrated that *BRAF* V600E mutations were associated with older age (*p* < 0.001) [27]. This discrepancy may arise because our findings are based on a population of thyroid nodules. Additionally, a meta-analysis reported an association between the *BRAF* mutation and male gender [28].

In this study, the frequency of the *BRAF* V600E mutation in malignant thyroid nodules was 70.22% (158/225), which was consistent with the results reported in the Chinese population (63.6∼87.7%) and higher than that in the Western population (30∼70%) [8, 29, 30]. Six cases with *BRAF* mutations were confirmed as benign nodules, possibly because the patients were in the early stages of the disease. A prospective longitudinal study has reported that 39 patients with *BRAF* mutations confirmed as benign nodules at the beginning were all identified as malignant after 3 years of follow-up [31]. This demonstrates that the *BRAF* gene can predict the emergence of malignancy in benign thyroid nodules with high specificity. The high prevalence of *BRAF* V600E in the Chinese population may be attributed to abnormal iodine intake in the daily diet. An epidemiological study in China has demonstrated that the frequency of *BRAF* V600E mutations is higher among individuals with high iodine intake compared to those with normal iodine intake [32]. This suggests that iodine intake could be a risk factor for the occurrence of *BRAF* V600E mutations and the development of PTC. The population in this study primarily resides along the southeast coast of China, where iodine intake is relatively high, potentially contributing to the increased frequency of *BRAF* V600E mutations. A similar study conducted in Korea found that both low and excessive iodine intake may serve as significant risk factors for the development of *BRAF* mutations in the thyroid, thereby posing risks for the development of PTC in iodine-adequate areas [33]. In the context of iodine deficiency, there is an accelerated proliferation of follicular cells in response to elevated serum TSH levels, rendering them more susceptible to genetic alterations [34, 35]. Furthermore, retrospective studies have demonstrated that iodine intake may not have an impact on *BRAF* mutations in the thyroid and thus is not considered a risk factor for the development of PTC [36–38]. Consequently, further exploration is needed to understand the molecular mechanisms underlying the varying prevalence of *BRAF* V600E mutations among different populations.

The overall incidence of *TERT* mutations in this study was only 1.69% (14/826), which is lower than the rates reported in previous studies [21]. Eight (57.14%) patients exhibited concurrent *BRAF* V600E and *TERT* mutations. A similar association was observed in Chinese PTC patients, with a higher prevalence (94.7%) of coexisting mutations [27]. A meta-study reported that patients with coexisting mutations exhibit more clinical aggressiveness and have a poorer prognosis compared to those with *BRAF* or *TERT* mutations alone [39]. The potential mechanism of the synergistic effect of *BRAF* V600E and *TERT* promoter mutations on PTC invasion and prognosis may be attributed to the upregulation of several ETS transcription factors induced by BRAF, leading to increased TERT expression [40].

*RAS* mutations (*NRAS*, *HRAS*, and *KRAS*) with a prevalence of 9.32% (77/826) were the second most common mutations in this study, followed by *BRAF* V600E. Notably, *RAS* gene mutations have limited diagnostic value, as they can be found in adenomas, low-grade malignancies, and highly malignant tumors. Medici et al. addressed that five cases of *RAS*-*positive* thyroid nodules were observed with no significant nodule growth or other suspicious sonographic features during the mean 5-year follow-up period [10]. A similar phenomenon of some other gene mutations has been noted in recent literature. For instance, with an average follow-up period of 1.77 years, it is found that thyroid nodules with *PTEN* mutation are mostly benign and unlikely to exhibit rapid growth [41]. In addition, *TSHR*, *TTN*, *PIK3CA*, and *EIF1AX* mutations in thyroid nodules categorized as indeterminate are uncommon and are typically verified as benign [42].

Consistent with the previous report, the prevalence of *RET* fusion in thyroid cancer is 3.11% (7/225), while the occurrence of *NTRK3* fusion in thyroid cancer is infrequent, with a frequency of 0.95% (2/225) in our study. The *RET* fusion has been reported as the most prevalent mutation in pediatric PTC, exhibiting a significant difference from the mutation profile observed in adult cases [43]. The fusion of *ETV6-NTRK3* has been documented to have a strong correlation with the prognosis and recurrence of thyroid tumor [44]. Recently, testing for *NTRK* fusion genes has gained increasing attention because of their potential for therapeutic targeting. Tumors with *NTRK* fusion genes have shown sensitivity to TRK inhibitors, such as larotrectinib and entrectinib, which have demonstrated good tolerability and effectiveness [18].

Considering the diagnostic value of genetic mutations, *BRAF* V600E, *TERT*, and gene fusions have been incorporated into the 6-gene test panel to assist in the diagnosis of benign and malignant nodules. Compared to the 22-gene mutation assay analysis, the diagnostic performance of the 6-gene test panel demonstrated superior diagnostic value with higher specificity (from 74.03% to 89.61%), PPV (from 89.07% to 95.15%), and accuracy (from 83.97% to 86.26%) (Table 6). In this study, 23.79% (187/786) FNA samples were categorized as indeterminate, and only 25.67% (48/187) of the patients underwent surgical treatment, of which 62.50% (30/48) were categorized as malignant based on surgical pathology. A significant proportion of patients (about 74.33%) with indeterminate nodules choose not to undergo surgery. Han et al. reported that an 18-gene panel used in the evaluation of indeterminate thyroid nodules had a sensitivity of 76.2% and specificity of 66.7% for diagnostic performance [21]. In this cohort, the diagnostic performance of the 6-gene test panel for indeterminate thyroid nodules was better than what is reported in the literature. The sensitivity, specificity, NPV, PPV, and accuracy of the panel were 83.33%, 88.89%, 76.19%, 92.59%, and 85.42%, respectively (Table S3). Compared to the DNA-RNA classifier (sensitivity of 88.4%, specificity of 53.8%, PPV of 84.4%, and NPV of 61.5%), the sensitivity, specificity, PPV, and NPV of the 6-gene test panel were higher [45]. Nevertheless, the PPV and specificity of the panel were higher, and the NPV was lower than those of commercial ThyroSeq v3 (sensitivity of 96.9%, specificity of 35.7%, PPV of 63.3%, and NPV of 90.9%) [46]. The discrepancy in the diagnostic performance may be due to differences in usage scenarios. ThyroSeq v3 was used to rule out malignancy in indeterminate thyroid nodules due to the high sensitivity and NPV [47]. More importantly, with the higher PPV and the specificity, the 6-gene test panel as “rule in” tests was used to identify malignancy in indeterminate nodules and aid in making decision for surgery.

According to the latest Bethesda Thyroid Cytopathology Reporting System, samples in Bethesda V are also recommended for molecular testing. For the samples in Bethesda III, IV, and V, the diagnostic performance of the 6-gene test panel exhibited better than the 22-gene mutation assay analysis in terms of specificity (increasing from 77.78% to 88.89%) and PPV (increasing from 94.12% to 96.77%) (Table S4). Approximately 155 patients in Bethesda III, IV, and V will require surgery based on diagnostic results and the PPV of the 6-gene test panel, which is higher than the number that have undergone surgery. This is the value of the “rule in” test. The 6-gene test panel showed an acceptable ability to discriminate malignant among thyroid nodules, with an AUC of 0.87. Reducing the number of targets to test could effectively decrease DNA/RNA amount and sequencing data size required, thereby lowering the cost of NGS testing and improving diagnostic efficiency. Moreover, minimization of the testing could even facilitate the development of other detection methods such as qPCR assays, which may be more flexible and advantageous in specific scenarios considering health economics, report turnaround time (TAT), and other requirements.

Twenty two NGS-negative cases in FNA samples were subsequently diagnosed as malignant cancer after surgery. The disparity in results between NGS testing and surgical pathology may be attributed to sampling experience. If the sampling area is inaccurate or if too few tumor cells are collected, particularly in the case of tiny thyroid nodules, the accuracy of the NGS results might be affected. In a clinical setting, the selection of surgery based on ultrasound characteristics, FNA cytology, clinical symptoms, and patient willingness is not sufficient. Molecular testing may provide valuable assistance in the decision-making process.

However, there are certain limitations to this study. The insufficient number of samples, particularly for indeterminate nodules with both cytologic and surgical pathological results, as well as the lack of follow-up data for some NGS-positive samples, led to bias in evaluating the diagnostic value of NGS testing. The thyroid cancer FNA samples included in this study primarily consist of PTC, while rare subtypes such as FTC, PDTC, MTC and ATC are absent. Additionally, there are deficiencies in the collection of clinical information regarding the samples, such as iodine intake. These limitations hinder the ability to conduct in-depth research on the factors influencing the development of thyroid cancer and to determine whether these factors are associated with genetic mutations.

In the future, we will continue to follow these patients for 3∼5 years, monitor the prognosis, and update the data to analyze the relationship between gene mutations and the development and prognosis of thyroid cancer. Currently, the samples are collected from the southeastern coastal regions, and we plan to conduct multicenter clinical trials in the future that will encompass different regions and include a broader range of thyroid cancer subtypes. This approach will allow for a more comprehensive evaluation of the performance of the NGS panel. Throughout this process, promising novel molecular markers, including gene mutations, gene fusions, methylation, etc., may be identified, which could enhance the sensitivity and specificity of diagnosis. Ultimately, the research aims to provide clinical practice with valuable tools for the entire continuum of care, from diagnosis and treatment to monitoring and prognosis.

## 5. Conclusion

In summary, a validated molecular test based on a comprehensive panel of DNA mutations and RNA fusions is utilized to distinguish histopathological malignant nodules from cytologically indeterminate nodules. The 22-gene mutation assay showed a genetic profile of thyroid nodules in the Chinese populations with a high frequency of *BRAF* and *RAS* mutations. The results of the 6-gene test panel (*BRAF* V600E, *TERT* mutations, and gene fusions) demonstrated excellent diagnostic performance with high specificity and PPV in diagnosing thyroid nodules. For the diagnosis of the indeterminate nodules and SUSP nodules, the 6-gene test panel as a “rule in” test showed better clinical value with a higher PPV. Nevertheless, additional large-scale validation, particularly in prospective cohorts, is necessary to enhance the accuracy of diagnosing and treating thyroid nodules.

## Figures and Tables

**Table 1:** Characteristics of the patients.

Category		Number of samples (*n* = 826)
Sex	Male	142
	Female	684

Age	15∼47	360
	48∼63	358
	≥ 64	108

**Table 2:** Cytology and surgical pathology results of samples.

Cytology	Number of samples (*n* = 826)	Surgical pathology		
Bethesda		Benign	Malignant	Unknown
I	45	0	2	43
II	250	4	3	243
III	157	15	29	113
IV	30	3	1	26
V	129	0	40	89
VI	17	0	7	10
No cytology	198	55	143	0

**Table 3:** Relationship between genetic mutations with gender and age.

	Genetic alterations		*p* value	*BRAF* V600E		*p* value	*TERT* promoter mutations		*p* value	Gene fusions		*p* value
	Positive	Negative		Positive	Negative		Positive	Negative		Positive	Negative	
Number of patients	409 (49.52%)	417 (50.48%)		300 (36.32%)	526 (63.68%)		14 (1.69%)	812 (98.31%)		24 (2.91%)	802 (97.09%)	
Gender			0.770			0.785			0.075			0.784
Female	341	343		247	437		9	675		21	663	
Male	68	74		53	89		5	137		3	139	
Age (years)			< 0.01			< 0.001			< 0.01			< 0.05
Mean ± SD	47.56 ± 12.28	50.37 ± 12.78		46.95 ± 11.99	50.13 ± 12.81		62.07 ± 12.40	48.75 ± 12.50		43.79 ± 10.20	49.13 ± 12.65	
95% CI	46.37 − 48.75	49.14 − 51.60		45.59 − 48.31	49.03 − 51.23		55.57 − 68.57	47.89 − 49.61		39.71 − 47.87	48.25 − 50.01	

**Table 4:** Distribution of gene alterations in the samples.

	Surgical pathology				Cytology						
	Total	Malignant	Benign	Unknown	I	II	III	IV	V	VI	No cytology
*BRAF* V600E	281	147	6	128	5	30	49	0	95	11	91
*BRAF* V600E/*TERT* C228T	6	4	/	2	/	1	/	/	1	1	3
*BRAF* V600E/*TERT* C250T	2	2	/	/	/	/	1	/	/	/	1
*BRAF* V600E/*CCDC6-RET*	1	/	/	1	/	/	/	/	1	/	/
*BRAF* V600E/*NRAS* Q61R	1	1	/	/	/	/	1	/	/	/	/
*BRAF* V600E/*NRAS* Q61R/*AKT1*	1	1	/	/	/	/	1	/	/	/	/
*BRAF* V600E/*NRAS* Q61K	1	/	/	1	/	/	/	/	/	1	/
*BRAF* V600E/*TTN* S3536N	1	/	/	1	/	/	/	/	/	1	/
*BRAF*V600E/*PIK3CA* H1047R	1	1	/	/	/	/	/	/	/	1	/
*BRAF* V600E/*PTEN* 802 2AD>T	2	1	/	1	/	/	/	/	/	1	1
*BRAF* V600E/*TSHR* R450H	2	1	/	1	/	/	/	/	1	/	1
*BRAF* V600E/*KRAS* GQ61GK	1	/	/	1	/	/	/	/	1	/	/
*TERT* C228T	2	1	/	1	/	1	/	/	/	/	1
*TERT* C228T/*ETV6-NTRK3*	1	/	1	/	/	/	/	/	/	/	1
*CCDC6-RET*	12	6	/	6	/	2	4	/	3	/	3
*NCOA4-RET*	2	1	1	/	/	/	/	/	/	/	2
*ETV6-NTRK3*	4	1	/	3	1	/	2	/	/	/	1
*TERT* C228T/*NRAS* Q61R	1	1	/	/	/	/	/	/	/	/	1
*TERT* C228T/*TERT* C250T/*NRAS* Q61R	1	1	/	/	/	/	/	/	//		1
*TERT* C228T/ *HRAS* Q61K	1	/	/	1	/	/	/	1	/	/	/
*NRAS* Q61R	40	3	5	32	2	19	6	1	6	/	6
*NRAS* Q61K	7	/	/	7	/	7	/	/	/	/	/
*TTN* S3536N	2	/	/	2	/	1	/	/	1	/	/
*PIK3CA* H1047R	1	/	1	/	/	/	/	/	/	/	1
*BRAF* K601E	2	/	1	1	/	/	1	/	/	/	1
*PTEN* 802-2A>T	2	/	/	2	/	/	2	/	/	/	/
*HRAS* Q61R	12	5	3	4	/	2	3	/	1	/	6
*HRAS* Q61K	3	/	1	2	/	2	/	/	/	/	1
*KRAS* Q61R	4	3	/	1	/	1	1	/	1	/	1
*KRAS* G12V	1	/	/	1	/	1	/	/	/	/	/
*TSHR* R450H	2		1	1	/	1	/	/	/	/	1
*NRAS* Q61R/*AKT1*	1	/	/	1	/	/	1	/	/	/	/
*EIF1AX* 338-1G>C	1	/	/	1	/	1	/	/	/	/	/
*EIF1AX* G8R	1	/	/	1	/	1	/	/	/	/	/
*KRAS* GQ61GK	2	/	/	2	/	2	/	/	/	/	/
*STRN-ALK*	2	2	/	/	/	/	/	/	/	/	2
*EML4-NTRK3*	1	1	/	/	/	/	/	/	/	/	1
*PAX8/PPARG*	1	/	/	1	/	/	/	1	/	/	/

*Note:* No distinction in sample types (FNA samples or FFPE samples).

**Table 5:** Information of samples with multiple mutations in this study.

Sample	Sex	Age	Bethesda	Pathology	Molecular testing
FNA-0042	Male	74	/	PTC	*BRAF* V600E/*TERT* C228T
FNA-0297	Female	57	/	PTC	*BRAF* V600E/*TERT* C228T
FNA-0318	Female	72	II	/	*BRAF* V600E/*TERT* C228T
FNA-0677	Female	38	V	/	*BRAF* V600E/*TERT* C228T
FNA-0771	Male	60	VI	PTC	*BRAF* V600E/*TERT* C228T
FFPE-002	Female	56	/	PTC	*BRAF* V600E/*TERT* C228T
FNA-0036	Female	69	/	PTC	*BRAF* V600E/*TERT* C250T
FNA-0068	Female	53	III	PTC	*BRAF* V600E/*TERT* C250T
FNA-0002	Female	46	V	/	*BRAF* V600E/*CCDC6*
FNA-0692	Female	50	III	PTC	*BRAF* V600E/*NRAS* Q61R
FNA-0807	Female	65	VI	/	*BRAF* V600E/*NRAS* Q61K
FNA-0755	Female	29	VI	/	*BRAF* V600E/*TTN* S3536N
FNA-0118	Female	37	VI	PTC	*BRAF* V600E/*PIK3CA* H1047R
FNA-0420	Female	52	VI	/	*BRAF* V600E/*PTEN* 802-2A>T
FNA-0830	Female	55	/	PTC	*BRAF* V600E/*PTEN* 802-2A>T
FNA-0084	Male	36	/	PTC	*BRAF* V600E/*TSHR* R450H
FNA-0184	Female	51	V	/	*BRAF* V600E/*TSHR* R450H
FNA-0855	Male	31	V	/	*BRAF* V600E/*KRAS* GQ61GK
FNA-0480	Female	56	/	Benign	*TERT* C228T/*ETV6-NTRK3*
FFPE-026	Female	82	/	PDTC	*TERT* C228T/*NRAS* Q61R
FNA-0774	Male	53	IV	/	*TERT* C228T/*HRAS* Q61K
FNA-0198	Female	45	III	/	*NRAS* Q61R/*AKT1*
FFPE-035	Male	81	/	ATC	*TERT* C228T/*TERT* C250T/*NRAS* Q61R
FNA-0330	Female	52	III	FTC	*BRAF* V600E/*NRAS* Q61R/*AKT1*

Abbreviations: ATC, anaplastic thyroid cancer; FTC, follicular thyroid carcinoma; PDTC, poorly differentiated thyroid carcinoma; PTC, papillary thyroid carcinoma.

**Table 6:** Diagnostic performance of NGS analysis in all surgical pathology results of samples.

	Surgical pathology		Sensitivity (%)	Specificity (%)	Accuracy (%)	PPV (%)	NPV (%)
	Malignant	Benign					
22-gene positive	163	20	88.11	74.03	83.97	89.07	72.15
22-gene negative	22	57					

6-gene positive	157	8	84.87	89.61	86.26	95.15	71.13
6-gene negative	28	69					

*Note:* 6-gene test panel includes *BRAF* V600E mutation, *TERT* mutation, and gene fusions.

**Table 7:** Diagnostic performance of the 6-gene test panel in Bethesda III, IV, and V.

	Surgical pathology		Sensitivity (%)	Specificity (%)	Accuracy (%)	PPV (%)	NPV (%)
	Malignant	Benign					
6-gene positive	60	2	85.71	88.89	86.36	96.77	61.54
6-gene negative	10	16					

## Data Availability

The data used to support the findings of this study are available from the corresponding author upon request.

## Supporting Information

Table S1. Relationship between genetic mutations with nodule size.

Table S2. Distribution of gene alterations in the FFPE samples.

Table S3. Diagnostic performance of the 6-gene test panel in Bethesda III and IV (indeterminate thyroid nodules).

Table S4. Diagnostic performance of 22-gene mutation assay in Bethesda III, IV, and V.
