# Supplementary material for: Molecular Diagnosis of Thyroid Nodules Using Next-Generation Sequencing in the Chinese Population
Source: Int J Endocrinol. 2025 Jun 20;2025:7728360. doi: 10.1155/ije/7728360 (PMC12204740; doi:10.1155/ije/7728360)
Supplement: Supporting Information — Additional supporting information can be found online in the Supporting Information section. [file 7728360.f1.docx]

**Supporting Information**

**Table S1** Relationship between genetic mutations with nodule size.

|  | Genetic alterations | | p Value | *BRAF* V600E | | p  Value | *TERT* promoter mutations | | p Value | Gene fusions | | p Value |
| --- | --- | --- | --- | --- | --- | --- | --- | --- | --- | --- | --- | --- |
|  | Positive | Negative |  | Positive | Negative |  | Positive | Negative |  | Positive | Negative |  |
| Number of patients | 349  (53.28%) | 306  (46.72%) |  | 266  (40.61%) | 389  (59.39%) |  | 7  (1.07%) | 648  (98.93%) |  | 19  (2.90%) | 636  (97.10%) |  |
| Mean size （mm) | 10.26±8.41 | 14.48±12.39 |  | 8.99±7.02 | 14.45±12.08 |  | 15.13±10.81 | 12.20±10.67 |  | 11.49±8.24 | 12.25±10.74 |  |
| Median quartiles （mm） | 8 (5.4-11) | 9 (5.2-20) |  | 7 (5.1-10) | 9 (5.7-20) |  | 11.2(9.5-13.5) | 8 (5.2-15) |  | 9(7.1-13.85) | 8 (5.2-15) |  |
| ≥5 mm | 286 | 247 | 0.69 | 214 | 319 | 0.62 | 7 | 526 | 0.36 | 16 | 517 | 1 |
| <5 mm | 63 | 59 |  | 52 | 70 |  | 0 | 122 |  | 3 | 119 |  |

In this cohort, 655 nodule size were collected. According to the genetic mutations, the mean size, median and quartiles were counted.

**Table S2** Distribution of gene alterations in the FFPE samples

| Sample | Sex | Age | Pathology | Molecular testing |
| --- | --- | --- | --- | --- |
| FFPE-25 | Male | 70 | ATC | *TERT* C228T |
| FFPE-30 | Male | 78 | ATC | */* |
| FFPE-35 | Male | 81 | ATC | *TERT* C228T/*TERT* C250T/*NRAS* Q61R |
| FFPE-38 | Male | 64 | ATC | *NRAS* Q61R |
| FFPE-27 | Male | 55 | Hurthle cell tumor | */* |
| FFPE-29 | Female | 39 | Hurthle cell tumor | */* |
| FFPE-32 | Male | 40 | Hurthle cell tumor | */* |
| FFPE-33 | Male | 62 | Hurthle cell tumor | */* |
| FFPE-36 | Male | 53 | Hurthle cell tumor | */* |
| FFPE-01 | Female | 52 | MTC | */* |
| FFPE-03 | Male | 47 | MTC | */* |
| FFPE-04 | Female | 49 | MTC | */* |
| FFPE-10 | Male | 42 | MTC | *KRAS* Q61R |
| FFPE-12 | Female | 31 | MTC | */* |
| FFPE-15 | Female | 76 | MTC | */* |
| FFPE-16 | Female | 49 | MTC | */* |
| FFPE-18 | Male | 49 | MTC | */* |
| FFPE-21 | Female | 34 | MTC | */* |
| FFPE-23 | Male | 41 | MTC | */* |
| FFPE-24 | Female | 81 | MTC | *HRAS* Q61R |
| FFPE-40 | Female | 32 | MTC | / |
| FFPE-26 | Female | 82 | PDTC | *TERT* C228T/*NRAS* Q61R |
| FFPE-28 | Female | 71 | PDTC | *HRAS* Q61R |
| FFPE-31 | Male | 72 | PDTC | */* |
| FFPE-37 | Female | 36 | PDTC | */* |
| FFPE-39 | Female | 69 | PDTC | *NRAS* Q61R |
| FFPE-02 | Female | 56 | PTC | *BRAF* V600E/*TERT* C228T |
| FFPE-05 | Female | 38 | PTC | *BRAF* V600E |
| FFPE-06 | Female | 30 | PTC | */* |
| FFPE-07 | Female | 39 | PTC | *BRAF* V600E |
| FFPE-08 | Female | 36 | PTC | *BRAF* V600E |
| FFPE-09 | Male | 46 | PTC | *BRAF* V600E |
| FFPE-11 | Male | 51 | PTC | *BRAF* V600E |
| FFPE-13 | Female | 63 | PTC | *BRAF* V600E |
| FFPE-14 | Female | 29 | PTC | */* |
| FFPE-17 | Female | 32 | PTC | *CCDC6* |
| FFPE-19 | Male | 46 | PTC | *BRAF* V600E |
| FFPE-20 | Female | 39 | PTC | *BRAF* V600E |
| FFPE-22 | Female | 55 | PTC | *BRAF* V600E |
| FFPE-34 | Female | 70 | PTC | *BRAF* V600E |

**Table S3.** Diagnostic performance of the 6-gene test panel in Bethesda Ⅲ and Ⅳ (indeterminate thyroid nodules).

|  | Surgical Pathology | | Sensitivity | Specificity | Accuracy | PPV | NPV |
| --- | --- | --- | --- | --- | --- | --- | --- |
|  | Malignant | Benign |  |  |  |  |  |
| 6-gene positive | 25 | 2 | 83.33% | 88.89% | 85.42% | 92.59% | 76.19% |
| 6-gene negative | 5 | 16 |  |  |  |  |  |

**Table S4**. Diagnostic performance of 22-gene mutation assay in Bethesda Ⅲ, Ⅳ and Ⅴ.

|  | Surgical Pathology | | Sensitivity | Specificity | Accuracy | PPV | NPV |
| --- | --- | --- | --- | --- | --- | --- | --- |
|  | Malignant | Benign |  |  |  |  |  |
| 22-gene positive | 64 | 4 | 91.43% | 77.78% | 88.64% | 94.12% | 70.00% |
| 22-gene negative | 6 | 14 |  |  |  |  |  |
